# Pharmacogenetic information in Swiss drug labels – a systematic analysis

**DOI:** 10.1038/s41397-020-00195-4

**Published:** 2020-10-17

**Authors:** C. Jeiziner, K. Suter, U. Wernli, J. M. Barbarino, L. Gong, M. Whirl-Carrillo, T. E. Klein, T. D. Szucs, K. E. Hersberger, H. E. Meyer zu Schwabedissen

**Affiliations:** 1 Pharmaceutical Care Research Group, Department of Pharmaceutical Sciences, University of Basel, Basel, 4001 Switzerland; 2 European Center of Pharmaceutical Medicine, Faculty of Medicine, University of Basel, Basel, 4056 Switzerland; 3 Department of Biomedical Data Sciences, Stanford University, Stanford, CA 94305 USA; 4 Department of Medicine, Stanford University, Stanford, CA 94305 USA; 5 Biopharmacy, Department of Pharmaceutical Sciences, University of Basel, Basel, 4056 Switzerland

**Keywords:** Drug regulation, Predictive markers, Gene therapy

## Abstract

Implementation of pharmacogenetics (PGx) and individualization of drug therapy is supposed to obviate adverse drug reactions or therapy failure. Health care professionals (HCPs) use drug labels (DLs) as reliable information about drugs. We analyzed the Swiss DLs to give an overview on the currently available PGx instructions. We screened 4306 DLs applying natural language processing focusing on drug metabolism (pharmacokinetics) and we assigned PGx levels following the classification system of PharmGKB. From 5979 hits, 2564 were classified as PGx-relevant affecting 167 substances. 55% (*n* = 93) were classified as “actionable PGx”. Frequently, PGx information appeared in the pharmacokinetics section and in DLs of the anatomic group “nervous system”. Unstandardized wording, appearance of PGx information in different sections and unclear instructions challenge HCPs to identify and interpret PGx information and translate it into practice. HCPs need harmonization and standardization of PGx information in DLs to personalize drug therapies and tailor pharmaceutical care.

## Introduction

“One size fits all” is the common strategy of dose-finding studies and consequently, the standard of drug therapy. However, drug therapies may fail, and/or may induce considerable adverse drug reactions (ADRs). The influence of patients’ genetic predispositions on drug response has been studied over decades and therefore, pharmacogenetics (PGx) is gaining attendance in patient-centered research and personalized medicine [1–3].

For the translation of PGx information into clinical decisions, health care professionals (HCPs) have to consider drug-gene interactions (DGIs) in addition to drug-drug interactions (DDIs). Similar to DDIs, not all DGIs require an intervention. The level of actionability depends on both the genetic variant of an enzyme and the metabolized substrate. Almost 100% of the population carry at least one actionable genetic variant [4, 5]. Consequently, it is expected that implementation of PGx into clinical decisions might be a strategy to reduce the substantial burden of ADRs [6], still representing a major concern in health care [7]. Considering the high number of drug-relevant genes and the multitude of available substances on the market, genetic variability potentially affects a large number of patients.

In this study, we focused on the drug label (DL), one of the first sources for HCPs to check for information on a drug. The Pharmacogenomics Knowledgebase (PharmGKB) [8] is an expert curated knowledgebase which collects and disseminates information on DGIs. The website (https://www.pharmgkb.org) is publicly available and supports researchers and clinicians in the interpretation of human genetic variation in relation to drug response. Information available includes prescribing information from clinical guidelines, curated pathways, pharmacogene summaries, annotations on associations between genetic variants and drug responses as reported in the literature, and DLs containing PGx information [9]. The PharmGKB has defined four PGx levels (see Table 1) to classify PGx information mentioned in DLs according to the potential for action [10].

**Table 1:** Definition of PGx levels of PharmGKB [10].

Nr.	PGx level	Definition
1	Testing required	The label states or implies that some sort of gene, protein, or chromosomal testing, including genetic testing, functional protein assays, cytogenetic studies, etc., should be conducted before using this drug. This requirement may only be for a particular subset of patients. PharmGKB considers labels that state the variant is an indication for the drug, as implying a test requirement. If the label states a test “should be” performed, this is also interpreted as a requirement.
2	Testing recommended	The label states or implies that some sort of gene, protein or chromosomal testing, including genetic testing, functional protein assays, cytogenetic studies, etc., is recommended before using this drug. This recommendation may only be for a particular subset of patients. PharmGKB considers labels that say testing “should be considered” to be recommending testing.
3	Actionable PGx	The label may contain information about changes in efficacy, dosage, metabolism or toxicity due to gene/protein/chromosomal variants or phenotypes (e.g., “poor metabolizers”). Or, the label may mention contraindication of the drug in a particular subset of patients with particular variants/genotypes/phenotypes. However, the label does not require or recommend gene, protein or chromosomal testing.
4	Informative PGx	1. The label contains information stating that particular gene/protein/chromosomal variants or metabolizer phenotypes do not affect a drug’s efficacy, dosage, metabolism, or toxicity. Or, the label states that particular variants or phenotypes affect a drug’s efficacy, dosage, metabolism or toxicity, but this effect is not “clinically” significant. OR 2. The label appears or appeared on the FDA Biomarker List but does not currently meet the requirements to be assigned as “Testing required”, “Testing recommended” or “Actionable PGx.” PharmGKB annotates every label that appears on the FDA Biomarker list, regardless of whether we would otherwise annotate the label.

Several groups have compared the information on PGx in DLs authorized by different agencies [11–14]. In the United States, the Food and Drug Administration (FDA) approves the DLs, and provides a table of pharmacogenomic biomarkers in DLs [15]. In Switzerland, Swissmedic approves all Swiss DLs before they become publicly available (www.swissmedicinfo.ch). The Swiss DL is organized in different sections with defined headings; however, no section is dedicated to PGx. For the DLs of Switzerland, no overview or comparison of PGx information in the DLs exists at this time. By analyzing the DLs, we will get an overview of the current state of PGx information helping us to identify inconsistencies and to suggest potential improvement for the future.

Accordingly, it was the aim of this project to provide a systematic analysis of the Swiss DL sections reporting PGx-relevant information on metabolizing enzymes and transporters as well as HLA risk alleles, to evaluate the instructions provided to HCPs on PGx information and finally, to compare the respective PGx level with those provided in DLs authorized by agencies of other countries.

## Methods

### Natural language processing (NLP)

**Fig. 1:**
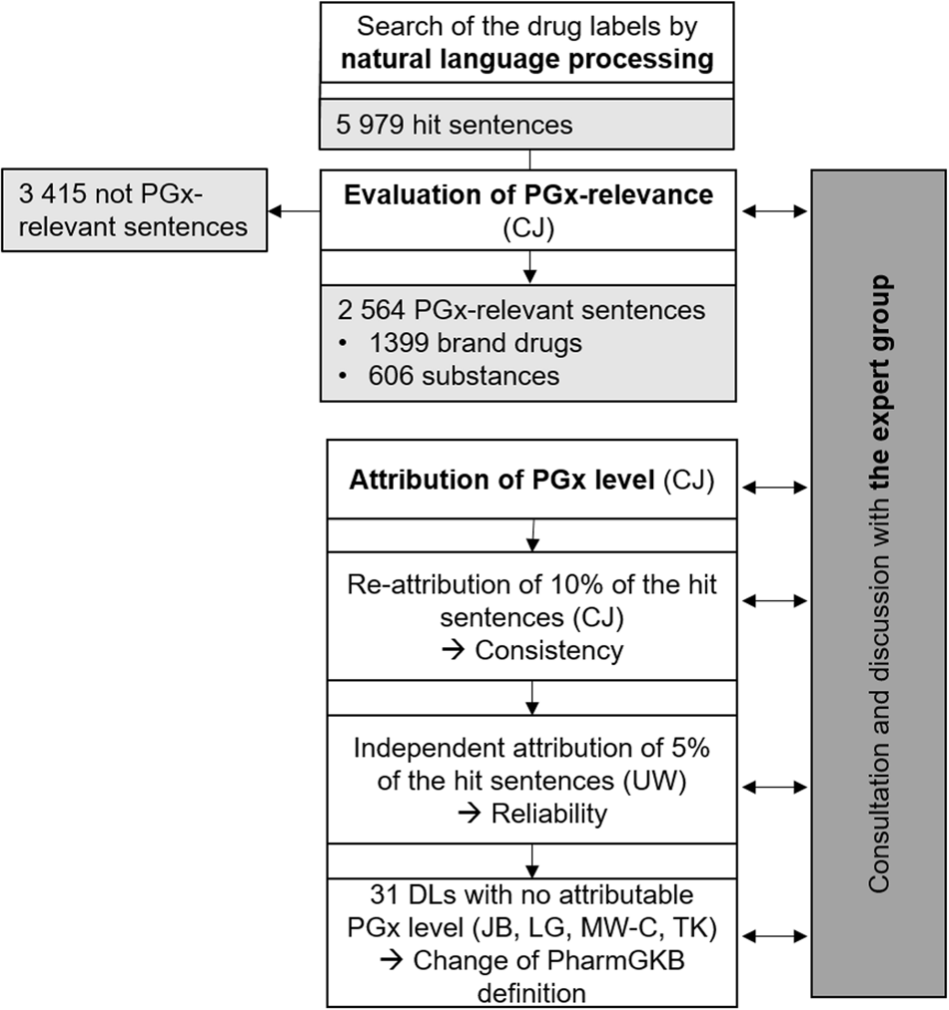
Flow chart depicting the process of analysis. The natural language processing was followed by evaluation of the PGx-relevance as well as the attribution of the PGx level, which was both conducted under consultation of the expert group.

We applied natural language processing (NLP). Terms used to search for PGx information within the DLs were gathered based on preliminary analysis of DLs (in German language), literature [16–18], and the AmiKoWeb website (https://amiko.oddb.org). The selected search terms to identify specific genes were related to genetic polymorphisms (defined as genetic variants with a prevalence of more than 1% in a population [3]) known to be involved in drug metabolism. An expert group (CJ, KS, KH, HMzS) selected 25 eligible word stems corresponding to 245 different search terms for the NLP (for details, see Supplementary Fig. 1). We used AmiKoWeb for the full-text search on 4th February 2019. All 4306 Swiss DLs available in German describing the 15,367 products on the Swiss market (including different dosages and package sizes) were screened for PGx information by NLP. The search identified 5979 hit sentences (corresponding to 606 chemical substances and 1399 different brand drugs) (Fig. 1). Supplementary Fig. 2 gives an overview of the primary NLP search.

### Evaluation of the identified sentences for PGx-relevance

We examined all hit sentences for PGx-relevance. Any information related to a genetic polymorphism of an enzyme known to be involved in drug metabolism or drug transport (pharmacokinetics) and any information on HLA risk types was considered PGx-relevant. We consequently excluded genetic mutations (prevalence <1%), disease-related gene defects (e.g., genetic hypercholesterolemia), disease-related chromosomal abnormalities (e.g., Philadelphia chromosome), nonhuman genetic factors (e.g., genotype of viruses), genes encoding proteins used for treatment selection (e.g., in oncology), and biomarkers related to a drug other than the referenced drug (e.g., in the case of an interaction).

### Classification of the PGx-relevant sentences

The classification of the sentences in the identified DLs was based on the PGx levels proposed by PharmGKB [10] (Table 1). If one sentence in one DL resulted in a higher PGx level compared to other sentences in the same DL, the highest annotated PGx level was considered in the analysis. After the first annotation of PGx levels, 10% of the sentences were reannotated to evaluate consistency and 5% were independently annotated by a second person to test reliability. After each step, the expert group was consulted. We translated the PGx-relevant sentences into English and submitted them to PharmGKB. Final discrepancies were solved in collaboration with experts of the PharmGKB group (Fig. 1).

We checked the PGx-relevant sentences of the same chemical substances (indicated by the ATC code level 1) and in case of multiple brand products with the same text in the DL, we defined one reference DL (refDL); either we selected the original product (brand name) or we arbitrarily choose the first generic drug in the list. We refer to Supplementary Table 1 for details on the refDLs. We analyzed the PGx-relevant sentences and the refDLs by the section where the PGx information was located, the anatomic groups (indicated by the ATC code level 1 of the corresponding substance), and the biomarker mentioned in the concerned PGx-relevant sentence, respectively.

### Annotations entered into the PharmGKB knowledgebase

PharmGKB applied their process of quality control to the translated DLs, annotated and entered them into the PharmGKB knowledgebase (for details see Supplementary Fig. 3).

### Comparison of PGx levels with those of other regulatory authorities

We conducted a comparative analysis of the annotated PGx levels available on PharmGKB of selected DLs with US Food and Drug Administration (FDA), European Medicines Agency (EMA), Health Canada/Santé Canada (HCSC), and Pharmaceuticals and Medical Devices Agency (PMDA), Japan. For the quantitative analysis, the PGx level was coded with points, with an increasing number of points for the severity of the PGx level, resulting in 1 point for “informative PGx” and 4 points for “testing required.”

## Results

### PGx-relevant information in Swiss Drug Labels searched by NLP

**Fig. 2:**
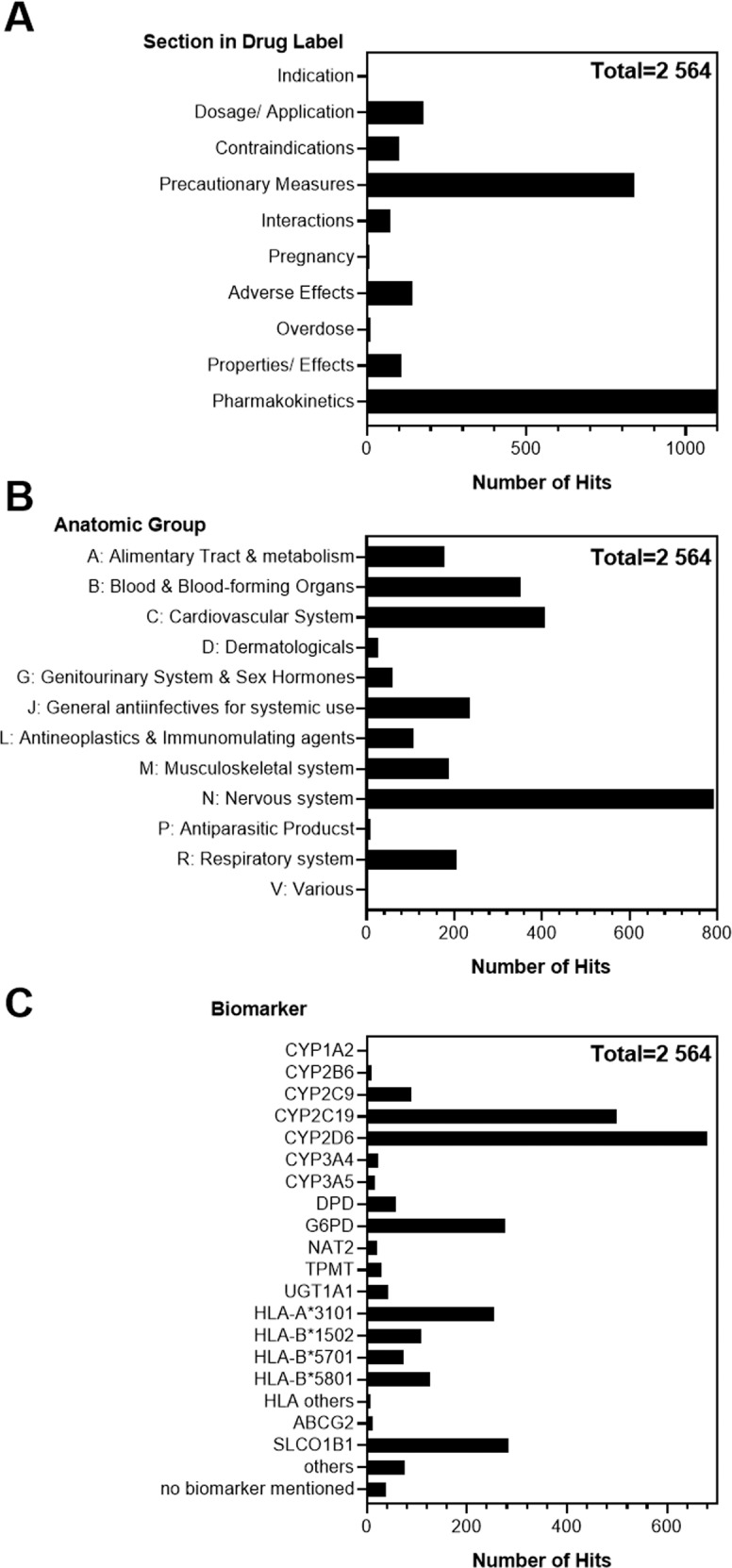
Analysis of the 2564 PGx-relevant sentences. Appearance in the different drug label sections (**A**), in the anatomic groups (indicated by the ATC code) of the drug described by the drug label (**B**) and of the biomarker mentioned in the sentences (**C**). Data shown are total number of PGx-relevant sentences per category.

From the 5979 identified hit sentences identified by the NLP search, 2564 sentences were classified as PGx-relevant. In total, 3415 sentences were excluded due to the lack of PGx-relevance. Most of the PGx-relevant sentences were part of the section on pharmacokinetics (*n* = 1110), followed by the precautionary measures section (*n* = 839). The other PGx-relevant sentences were distributed homogenously in the other sections (dosage/application, contraindications, interactions, adverse effects, or properties/effects). A small number of PGx-relevant sentences appeared in the sections on indication (*n* = 3), pregnancy (*n* = 7), or overdose (*n* = 10) (Fig. 2A). No PGx-relevant information appeared in any of the ten remaining sections, such as ability to drive or operate machines, preclinical data, or other hints.

Most of the PGx-relevant sentences were found in the ATC group “N: Nervous system” (*n* = 793), followed by “C: Cardiovascular system” (*n* = 408), and “B: Blood and blood building systems” (*n* = 352). The lowest number of PGx-relevant sentences appeared in the ATC group “V: Various” (*n* = 3). No PGx-relevant sentences were discovered in the ATC group “H: Systemic hormonal preparations” (Fig. 2B).

The PGx biomarker most frequently mentioned was the drug metabolizing enzyme CYP2D6 (*n* = 679), followed by CYP2C19 (*n* = 499). The drug transporter SLCO1B1 (OATP1B1), the enzyme glucose 6-phosphate dehydrogenase (G6PD), and the HLA-allele HLA-A*3101 were named in *n* = 254, *n* = 277, and *n* = 284 sentences, respectively. Overall, 76 PGx-relevant sentences referred to other biomarkers, e.g., IL28B. However, in 39 cases, PGx information was provided without mentioning any specific biomarker (Fig. 2C).

### Analysis of the reference drug labels (refDLs)

Based on the PGx-relevant sentences, we defined 167 refDLs. Almost in all cases (166 of 167) the DL of the generics contained the same text as the original product. For the ATC code L01BC02 (fluorouracil), we defined two refDLs, because the texts of the DLs of Efudix^®^ and Fluorouracil Labatec^®^ differed in information. Of the defined refDLs, there were 17 combination products where PGx information was the same as for the mono products of each component. Therefore, these refDLs were not annotated separately. Moreover, there were four products (carbamazepine, escitalopram, fluorouracil, and codeine/acetaminophen) addressing more than one biomarker in the PGx-relevant sentences with different PGx levels.

**Fig. 3:**
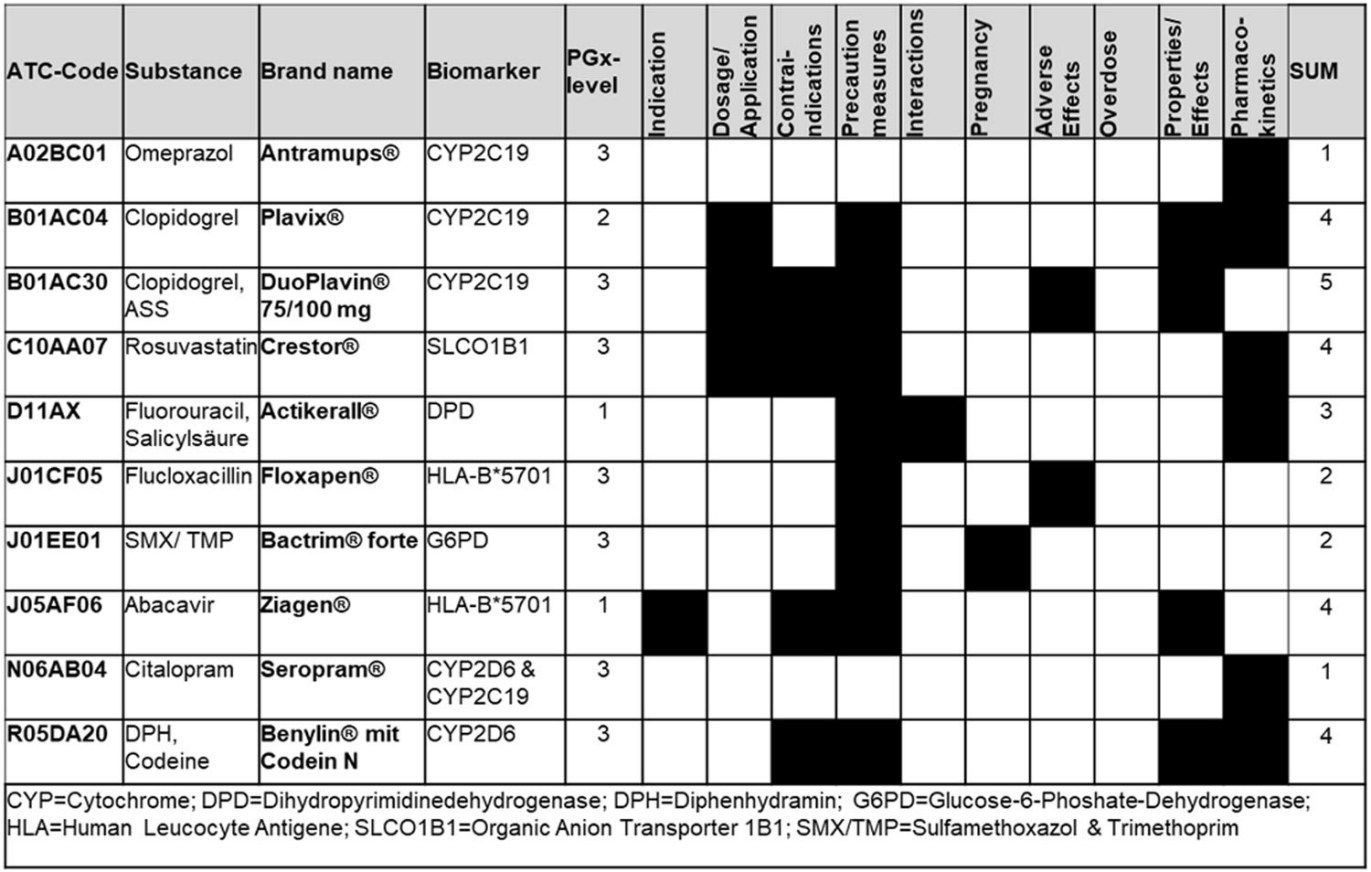
Drug label (DL) sections with PGx information. Ten examples of DLs indicating the different DL sections in which the information on PGx is given. The corresponding section is marked with black color. The last column shows the sum of sections that specify PGx information.

The PGx information of the refDLs was identified in 10 out of 20 different sections in the DL. One example, where PGx-relevant information is given in multiple sections namely “indication,” “precautionary measures,” “contraindications,” and “properties/effects” is abacavir (Ziagen^®^); for further examples see Fig. 3 (for details on all substances see Supplementary Table 1).

**Fig. 4:**
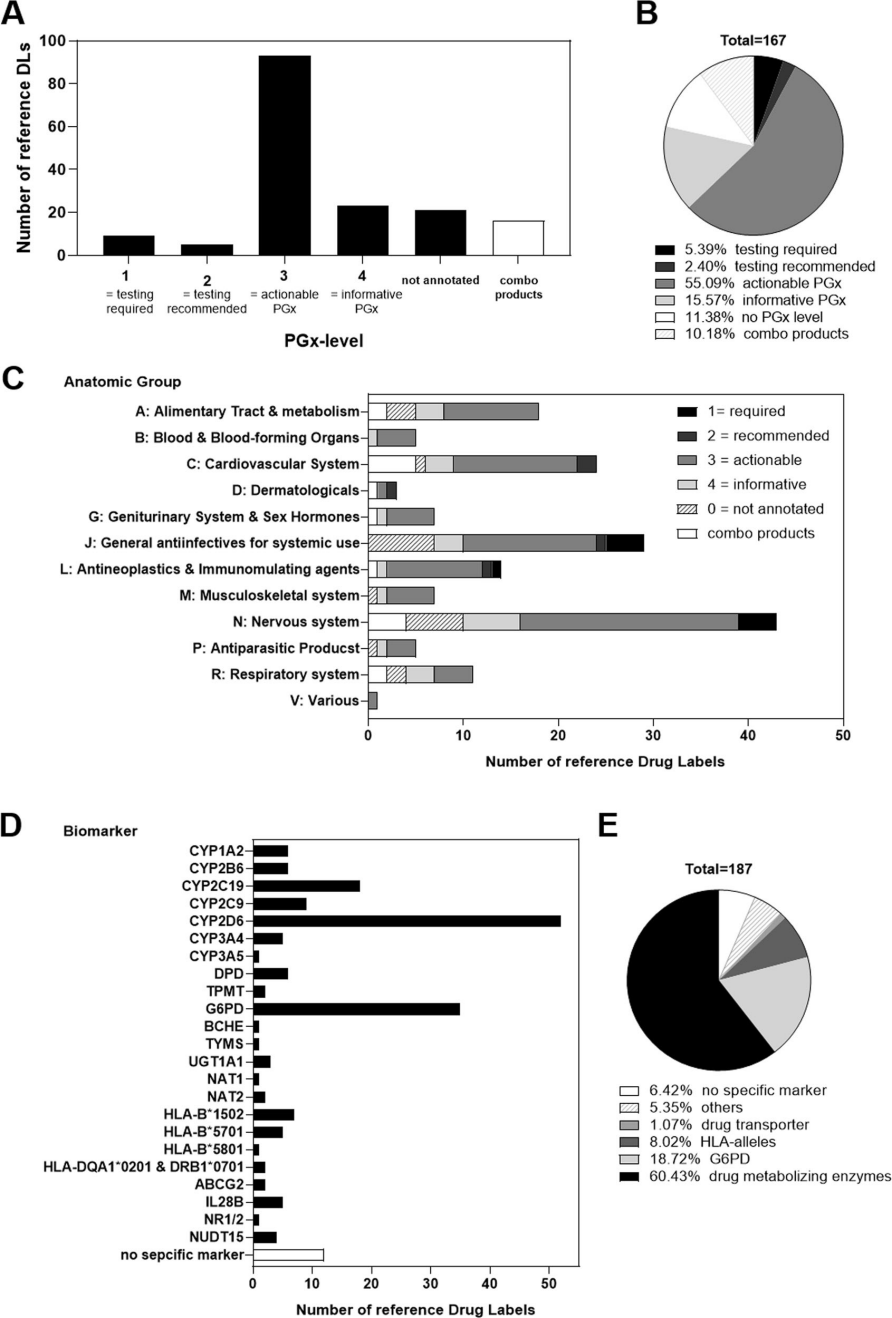
Analysis of the reference drug labels (refDLs). Number of reference drug labels per PGx level (**A**, **B**), per anatomic group and the assigned PGx levels (**C**), and per biomarker (**D**, **E**).

Most of the refDLs (*n* = 92 substances, 55%) were assigned to PGx level 3 “actionable PGx” and PGx level 4 “informative PGx” (*n* = 26, 16%). Only 9 (5%) or 4 (2%) DLs were assigned to PGx level 1 “testing required,” or PGx level 2 “testing recommended,” respectively. In total, 19 DLs (11%) could not be classified using the original definition of the PGx levels, as the information given did not meet the criteria proposed by PharmGKB, and 17 (10%) refDLs on combination products reported the same information as the mono product (Fig. 4A). Summarizing the PGx level annotated refDLs in anatomic groups (ATC code of level 1), revealed that PGx level 3 appeared most frequently (Fig. 4B). The anatomic group “J: general anti-infectives for systemic use” represented an exception as it contained abacavir (including three combination products), all labeled with PGx level 1. The same PGx level was attributed to carbamazepine, oxcarbazepine, codeine, and tetrabenazine as well as fluorouracil in the anatomic groups “N: Nervous system” and “L: antineoplastic and immunomodulating agents”, respectively (Fig. 4C).

Looking at the specific biomarkers in the refDLs (Fig. 4D), CYP2D6 was most frequently mentioned (*n* = 52), followed by G6PD (*n* = 35). In total, 12 refDLs informed on PGx without mentioning a specific biomarker. Many refDLs stated two biomarkers, e.g., G6PD and CYP2D6 in Co-Dafalgan^®^, one accounting for acetaminophen and the other for codeine. Overall, biomarkers in drug-metabolizing enzymes predominated (Fig. 4E).

### Annotations entered into the PharmGKB knowledgebase

**Fig. 5:**
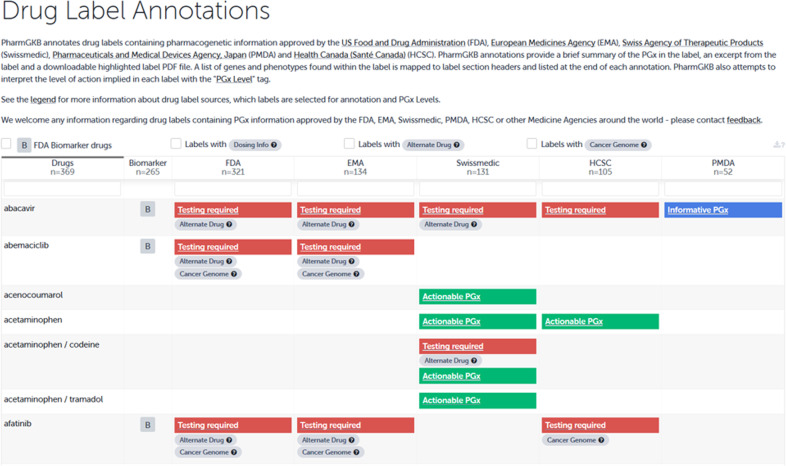
Excerpt of the Drug Label Annotations on the PharmGKB website. Since 08/07/2019 the Drug Label Annotations include excerpts of  the drug labels of the  Food and Drug Administration (FDA), the European Medicines Agency (EMA), the Swissmedic, the Health Care Service Cooperation (HSCS), and the Pharmaceutical and Medical Devices Agency (PMDA).

The extracts of the Swiss DLs were translated and entered into the PharmGKB knowledgebase on 22.10.2019 (https://www.pharmgkb.org/labelAnnotations) and resulted in 131 annotations (Fig. 5). In addition, the collaboration with PharmGKB led to a new definition for PGx level 4 “informative PGx.” The original definition of this category was “label mentioning a gene or protein involved in the metabolism or pharmacodynamics of the drug, with no information to suggest that variation in these genes/proteins leads to changes in drug response.” Due to difficulties in our primary analysis, we started a discourse with PharmGKB, which finally resulted in an adaptation of the definition of PGx level 4 (published on 08/07/2019).

### Comparison of PGx levels with those of other regulatory authorities

**Fig. 6:**
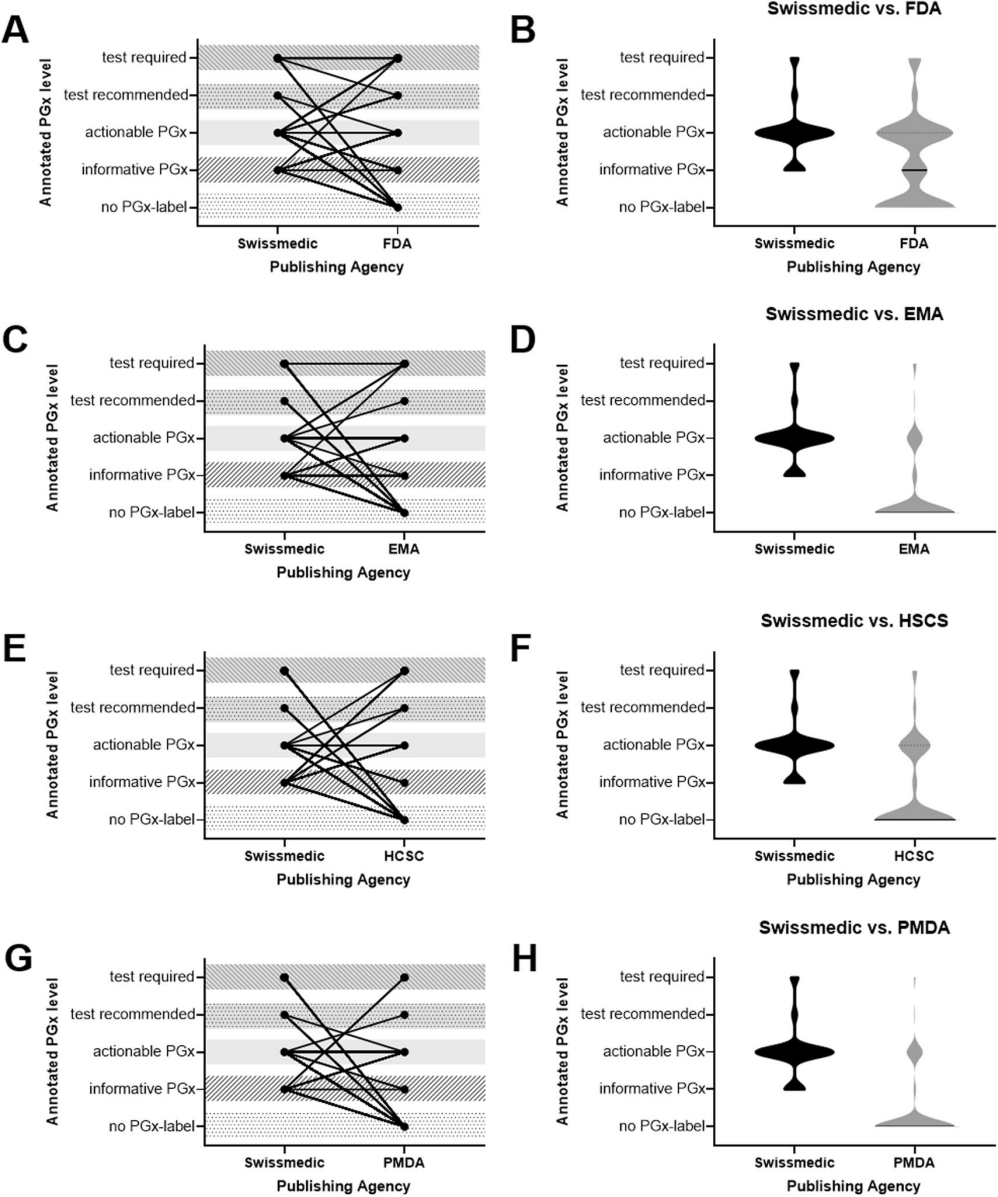
Comparative analysis of the attributed PGx levels of the Swiss DLs with those of other regulatory authorities, namely the Food and Drug Administration (FDA), the European Medicines Agency (EMA), the Health Care Service Cooperation (HSCS), and the Pharmaceutical and Medical Devices Agency (PMDA). Each drug was included in the schematic of **A**, **C**, **E**, and **G** linking its PGx level indicated by the respective DL of the publishing agency to visualize heterogeneity. In **B**, **D**, **F** and **H** the number of drugs in each category is indicated by the width of the violin.

We compared the assigned PGx levels of the 126 uploaded DLs of Swissmedic with those authorized by other regulatory authorities, and observed that the majority was rated as “actionable PGx”. This is also indicated, when determining a mean after translating the different categories into points. Here, the mean ± SD was 1.984 ± 0.693 (*n* = 126), 2.053 ± 0.831 (*n* = 76), 2.100 ± 0.8847 (*n* = 30), 2.178 ± 0.777 (*n* = 45), and 2.077 ± 0.688 (*n* = 26) points for Swissmedic, FDA, EMA, HCSC, and PMDA, respectively. However, the comparison also revealed that the PGx levels assigned (Fig. 6A, C, E, G) and the number of DLs reporting PGx-relevant information (Fig. 6B, D, F, H) were different. According to PharmGKB, “test required” was assigned to eight Swissmedic DLs, one EMA DL, and three FDA DLs. No PGx levels for these compounds were assigned to the DLs of HCSC or PMDA. From the FDA, eight DLs are rated as “test required”; while four of these DLs (gefitinib, rasburicase, tamoxifen, and ibrutinib) were rated differently in the Swissmedic DLs. Looking at the 126 DLs under consideration, all five regulatory authorities had a majority of DLs rated as “actionable PGx.” However, only the FDA have about the same number of DLs with “actionable PGx” as Swissmedic.

## Discussion

To our knowledge, this is the first NLP-based extraction of information related to PGx from the Swiss DLs. We focused genes involved in drug metabolism and transport (pharmacokinetics) and information on HLA risk alleles. We extracted 2564 PGx-relevant sentences, which corresponded to 167 chemical substances. Our analysis showed that 9.47% of all Swiss DLs (167 out of 1763 different ATC codes by 31st January 2019 [19]) mentioned PGx-relevant information. Most of this PGx information (55%) was classified as “actionable PGx”.

We identified the pharmacokinetics section as the prevailing section reporting on PGx. However, this particular section is – not only in the Swiss DLs, but also in those approved by other agencies – one of the last sections in a DL [20]. Therefore, it may be speculated that there is a risk that PGx information could be overseen by the HCPs. For some drugs coded with PGx level 1, the PGx-relevant information was located within the section on precautionary measures, which reports on genetic polymorphisms known to be associated with ADRs (especially in the case of HLA-associated ADRs, e.g., carbamazepine). Our findings are in line with those by Ehmann et al. [11] reporting that the pharmacokinetic and the precautionary measures section are most likely to state PGx information in DLs approved by the EMA. Other sections such as indications, dosage/use and contraindications rarely provide PGx information. In contrast to the study of Shimazawa et al. [21], we did not prioritize one section per DL, but we analyzed all sections mentioning PGx information.

In accordance with findings from other countries [14, 16, 17, 22], CYP2D6 was the most frequently mentioned biomarker in the Swiss DLs. This cytochrome P450 enzyme is known for its genetic variability with about 100 different alleles [23] resulting in the phenotypes of poor, intermediate, normal, and ultra-rapid metabolizer (UM) with a prevalence of 0.4–5.4%, 0.4–11%, 67–90%, and 1–21%, respectively [24]. Moreover, CYP2D6 is known to be involved in the metabolism of a wide range of commonly used drugs [25] including SSRIs [26], opioids [27–29], and tamoxifen [30]. The second most cited biomarker in the Swiss DLs was CYP2C19. This enzyme also affects a large number of drugs [25] including SSRIs [26, 29], opioids [29], and in particular the bioactivation of clopidogrel [31]. However, none of the Swiss DL contained the biomarker ABCB1, although the Swiss guideline on the treatment of unipolar depressive episodes recommends to test for selected genetic variants of ABCB1 (P-Glycoprotein) in patients taking antidepressants [32–34].

Although ABCB1 was not mentioned in the Swiss DLs, the anatomic group N (nervous system) dominated when analyzing PGx levels per anatomic group. This group contains antiepileptics (carbamazepine [35–37], oxacarbazepine [38], phenytoin [39, 40]), antidepressants such as SSRIs [29], or analgesics such as opioids [29]. The anatomic group N relates to various drugs where treatment is associated with more difficulties (e.g., therapy failure) compared to therapies of other anatomic groups. Indeed, Bschor et al. [41] and Muller et al. [42] assume that psychiatric patients would likely benefit from a PGx test prior to the therapy in order to avoid ADRs or therapy failure. The PGx-relevant information in anatomic group C mostly referred to statins [41] (e.g. fluvastatine [42]) and beta-blockers (e.g., metoprolol [43]). Almost all hits in the anatomic group B were related to clopidogrel, which is well-studied for the influence of genetic variability [31, 44, 45].

We identified nine (5%) refDLs with statements categorized as PGx level 1 and four (2%) refDLs as PGx level 2. For these drugs there is convincing evidence for the clinical benefit of PGx testing prior to treatment initiation. This may be explained by the severity of the potential ADRs [46]. For HCPs, the instruction in these DLs is clear. Accordingly, DLs containing information with PGx level 1 or 2 are most evident to handle, as clear recommendations on therapeutic consequences are given. The majority (55%) of the refDLs were classified as PGx level 3. They mention the influence of a genetic variant on drug efficacy or safety without recommending genetic testing. Here, the question is, how are HCPs supposed to handle this information. Should HCPs inform the patient, or simply take note of the information in case of ADRs or nonresponse? The predominance of PGx level 3 illustrates the insecurity which still dominates in the field of PGx. PGx level 4 was the second most applied PGx level (16%) for the Swiss DLs. The original definition of this category was adapted in a discourse with PharmGKB.

Overall, the presentation of PGx information is very heterogeneous; not only in terms of localization in the DL but also leading to different PGx levels and various associated recommendations. The information on PGx is often not precise and the presentation lacks a predefined structure. Similar findings have also been reported by Ehmann et al. (EMA) [11] and Shimazawa & Ikeda (US and Japan) [21]. By entering the extracts of 126 Swiss DLs on the PharmGKB website, we were able not only to make this information publically accessible but also comparable to the information approved by regulatory agencies of four other countries. The individual comparisons of the Swiss DLs with the DLs of the four different regulatory authorities listed in PharmGKB revealed a large heterogeneity not only in number of compounds with PGx information, but also in terms of assigned PGx levels for the available PGx information. Accordingly, there is a clear need for a standardized presentation with a well-defined structure.

Based on our analysis, there is a tendency toward more PGx testing (PGx level 1 and 2) in the Swiss DLs, compared to FDA or EMA. However, it has to be taken into account that the DLs of the EMA represent rather a general guidance, still enabling differences in the recommendations in national DLs. It has been recommended during the revision process of this manuscript that it should be considered to compare the Swiss DLs to the DLs published by the regulatory agencies of selected European countries. One country that would be suitable for such a comparison are the Netherlands, where guidelines on PGx are available and which appears to have an initiative for PGx implementation with the Dutch pharmacogenetic working group [47]. However, their DLs are only available in Dutch [48]. For Germany, we found a list of drugs published by VFA (Verein der Pharma-Forschenden) [49] with all substances which require or recommend PGx testing (analog to PGx level 1 and 2). In the context of pharmacogenotyping of genes relevant for pharmacokinetics, we are able to compare seven substances (see Supplementary Table 2).

Shekhani et al. [50] analyzed the concordance of the DLs of regulatory agencies with guidelines provided by CPIC/DPWG and revealed that out of 54 drugs with an actionable gene–drug interaction in the CPIC and DPWG guidelines, only 50% of the agencies described actionable PGx information in the DLs and they were in agreement in only 18% of the cases. We agree with Tan-Koi et al. [51] who suggested after a cross-sectional study of PGx associations in six different countries that there should be an international consensus for PGx presentation in DLs. Also Ehmann et al. [11] stated that the number of DLs mentioning PGx is steadily increasing and that a new legislation is necessary to support HCPs in the application of PGx information. In contrast to the FDA using subheadings on PGx, the current structure of the Swiss DLs does not support the incorporation of standardized PGx information.

### Limitations

We have to mention, that we searched for PGx-relevance with word stems concerning pharmacokinetics, thereby excluding information on pharmacodynamics. Our major concern was the inter-individual variability in drug metabolism, which is known to affect a great large number of patients in daily care. However, focusing on pharmacokinetics, we missed information on most oncological drugs, where genotyping is part of compound selection.

In contrast to most previous studies analyzing the DLs for PGx information by reviewers reading the DLs [11, 14], we applied an automated search by NLP. Of the total hit sentences identified by NLP, 43% contained PGx-relevant information. We consider NLP as a strength, even though we are aware of the effort which was necessary for the semantic standardization. As no predefined standardization for the presentation of PGx information in the Swiss DLs exists, the definition of word stems was challenging. In order to facilitate accessibility of DLs for NLP, standardization would be necessary.

During the attribution of PGx levels, we found DLs reporting on the same chemical substance, but stating different information. One reason for these discrepancies might be the different date of market admission. Moreover, a few DLs involved two or more biomarkers resulting in two PGx levels. Finally, some reference DLs inform on the influence of PGx on the drug’s efficacy or safety without mentioning a specific biomarker. These particular DLs were excluded from publishing on the website by PharmGKB, as they do not provide usable information for the HCP.

## Conclusion

The analysis of PGx information provided in Swiss DLs revealed large heterogeneity. PGx information varies not only in wording used to describe the information but also in the section, where the information appears. In addition, the instructions for clinical practice are rather vague. In summary, this makes the identification and the interpretation of PGx information difficult for HCPs. However, the predominance of PGx level 3 “actionable PGx” demonstrates that numerous actionable DGIs are existing, which could be considered in an optimized drug therapy. For their decision-making and patient counseling, HCPs depend on a supportive DL. Therefore, a specific section dedicated to PGx for the efficient identification of PGx information is favorable. Here, standardized language and well-structured, consistent presentation of PGx information within the DL would be required to facilitate accessibility (e.g., to NLP and then in a further step to clinical decision support systems). Finally, instructions on PGx testing should become more implicit, to support HCPs in personalizing drug therapies and tailoring pharmacotherapy.

## Supplementary information

Legends of Supplementary Material

Supplementary Figure 1

Supplementary Figure 2

Supplementary Figure 3

Supplementary Table 1

Supplementary Table 2
