# Supplementary material for: Pharmacogenetic information in Swiss drug labels – a systematic analysis
Source: Pharmacogenomics J. 2020 Oct 17;21(4):423–34. doi: 10.1038/s41397-020-00195-4 (PMC8292148; doi:10.1038/s41397-020-00195-4)
Supplement: Supplementary file 1 — Legends of Supplementary Material [file 41397_2020_195_MOESM1_ESM.docx]

# Legends of Supplementary Material

**Figure Legends**

Supplementary Figure 1: Word stems for the screening of the Swiss drug labels in German and English translation (combined with the Boolean operator “OR”); every selected word stem resulted in a list of various words, e.g. the word stem “genetisch” (engl. genetic) generated 11 different words or word combinations. The search included plural forms, different kinds of abbreviations, English terms, and even different ways of spelling as well as typing errors; terms, such as ‘Glucose-6-Phosphat-Dehydrogenase’, generated more than 10 different search terms.

Supplementary Figure 2: NLP search output in the MS Excel® file with Codeine example: The left part shows the automatic search (search term, substance name, brand drug name, ATC [Anatomical Therapeutic Chemical Classification System] code, section, corresponding sentence, and link to the online reference of the drug label) and the right part shows the manual data extraction (PGx-relevance, biomarker, and PGx level).

Supplementary Figure 3: Process of quality control by PharmGKB for the Swiss drug labels.

# Table Legends

Supplementary Table 1: Details on refDLs (anatomic group, ATC-code, substance, brand name, section, biomarkers, number of sections, marked sections, PGx level, link) 🡪*For a detailed legend, see MS Excel® file.*

Supplementary Table 2: Comparison Swiss drug labels and German VGA list
