# Supplementary figures and images for: Pharmacogenetic information in Swiss drug labels – a systematic analysis
Source: Pharmacogenomics J. 2020 Oct 17;21(4):423–34. doi: 10.1038/s41397-020-00195-4 (PMC8292148; doi:10.1038/s41397-020-00195-4)

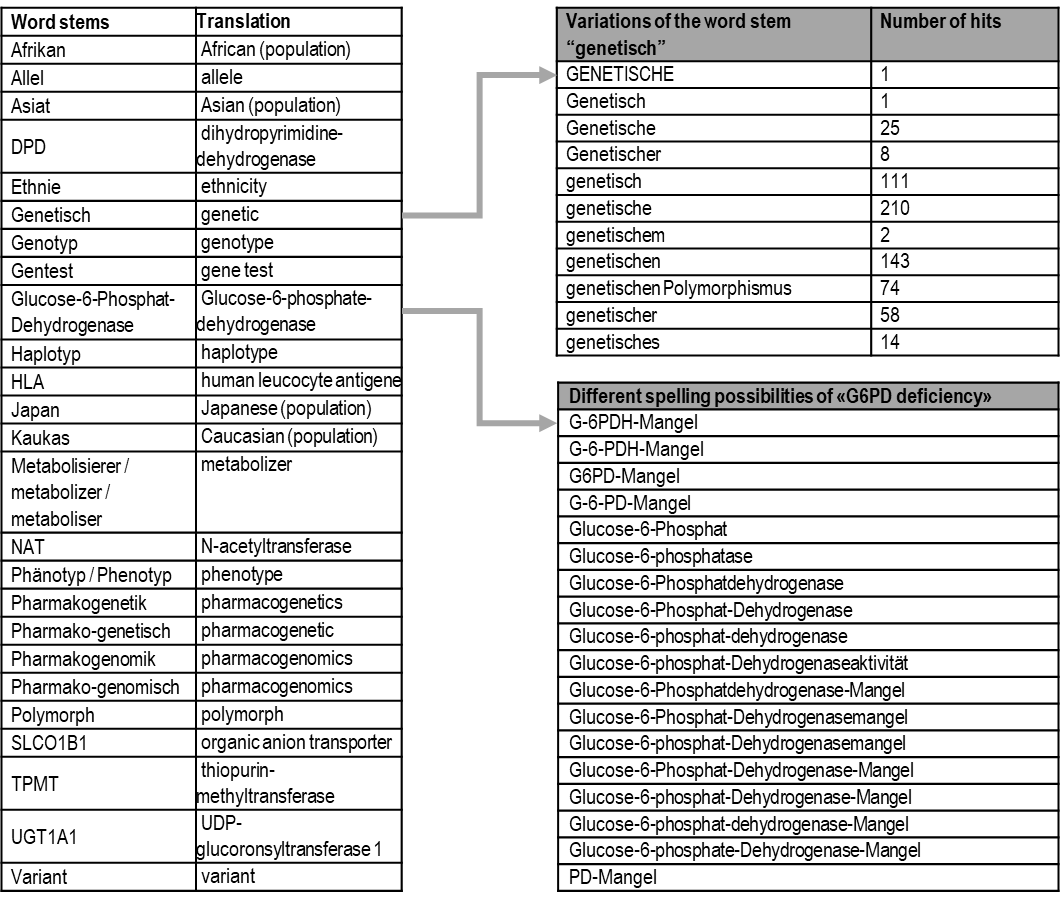

Supplement: Supplementary file 2 — Supplementary Figure 1 [file 41397_2020_195_MOESM2_ESM.tif]

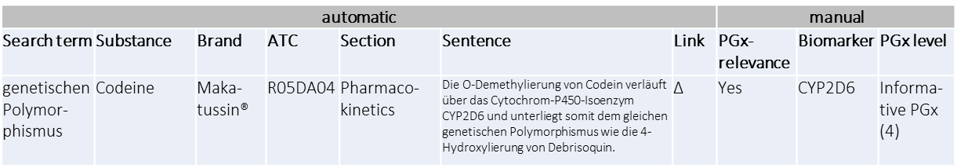

Supplement: Supplementary file 3 — Supplementary Figure 2 [file 41397_2020_195_MOESM3_ESM.tif]

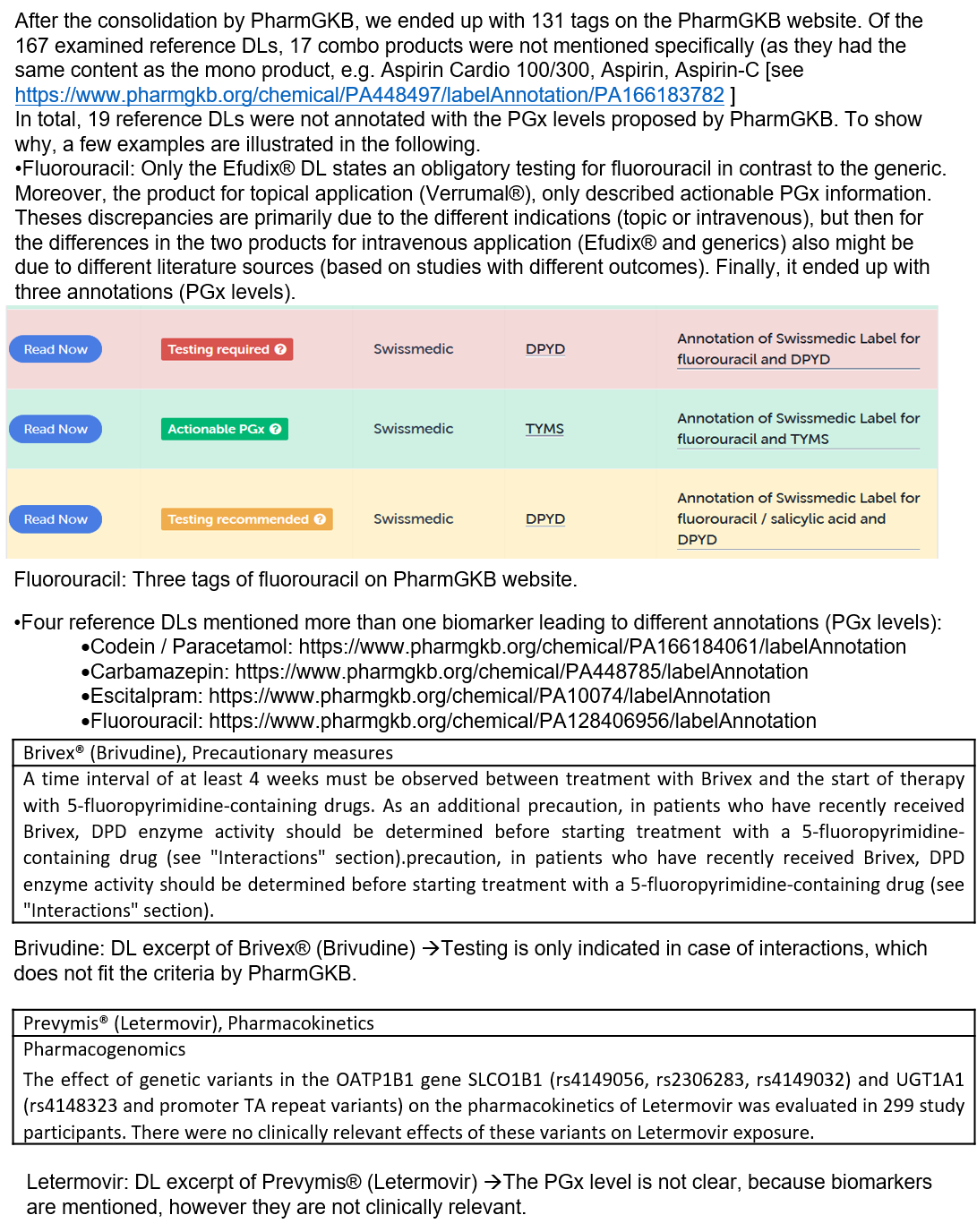

Supplement: Supplementary file 4 — Supplementary Figure 3 [file 41397_2020_195_MOESM4_ESM.tif]
